# Prognostic Value of Radiomic Features of ^18^F-FDG PET/CT in Patients With B-Cell Lymphoma Treated With CD19/CD22 Dual-Targeted Chimeric Antigen Receptor T Cells

**DOI:** 10.3389/fonc.2022.834288

**Published:** 2022-02-07

**Authors:** Yeye Zhou, Jihui Li, Xiaoyi Zhang, Tongtong Jia, Bin Zhang, Na Dai, Shibiao Sang, Shengming Deng

**Affiliations:** ^1^ Department of Nuclear Medicine, the First Affiliated Hospital of Soochow University, Suzhou, China; ^2^ Department of Nuclear Medicine, Changshu No. 2 People’s Hospital, Changshu, China; ^3^ Nuclear Medicine Laboratory of Mianyang Central Hospital, Mianyang, China; ^4^ State Key Laboratory of Radiation Medicine and Protection, Soochow University, Suzhou, China

**Keywords:** CAR-T, B-cell lymphoma, radiomics, PET/CT, prognosis

## Abstract

**Objective:**

In the present study, we aimed to evaluate the prognostic value of PET/CT-derived radiomic features for patients with B-cell lymphoma (BCL), who were treated with CD19/CD22 dual-targeted chimeric antigen receptor (CAR) T cells. Moreover, we explored the relationship between baseline radiomic features and the occurrence probability of cytokine release syndrome (CRS).

**Methods:**

A total of 24 BCL patients who received ^18^F-FDG PET/CT before CAR T-cell infusion were enrolled in the present study. Radiomic features from PET and CT images were extracted using LIFEx software, and the least absolute shrinkage and selection operator (LASSO) regression was used to select the most useful predictive features of progression-free survival (PFS) and overall survival (OS). Receiver operating characteristic curves, Cox proportional hazards model, and Kaplan-Meier curves were conducted to assess the potential prognostic value.

**Results:**

Contrast extracted from neighbourhood grey-level different matrix (NGLDM) was an independent predictor of PFS (HR = 15.16, p = 0.023). MYC and BCL2 double-expressor (DE) was of prognostic significance for PFS (HR = 7.02, p = 0.047) and OS (HR = 10.37, p = 0.041). The combination of NGLDM_Contrast_PET_ and DE yielded three risk groups with zero (n = 7), one (n = 11), or two (n = 6) factors (p < 0.0001 and p = 0.0004, for PFS and OS), respectively. The PFS was 85.7%, 63.6%, and 0%, respectively, and the OS was 100%, 90.9%, and 16.7%, respectively. Moreover, there was no significant association between PET/CT variables and CRS.

**Conclusions:**

In conclusion, radiomic features extracted from baseline ^18^F-FDG PET/CT images in combination with genomic factors could predict the survival outcomes of BCL patients receiving CAR T-cell therapy.

## Introduction

Diffuse large B-cell lymphoma (DLBCL) is the most common type of malignant lymphomas in adults, accounting for 30-40% of all non-Hodgkin lymphoma (NHL) worldwide, and DLBCL exhibits a higher prevalence in developing countries (1, 2). Indeed, a study from China has found that nearly half of the NHL cases are diagnosed as DLBCL (3). Chimeric antigen receptor (CAR) T-cell therapies targeting CD19 are a promising approach for the treatment of relapsed/refractory (R/R) B-cell malignancies. The complete remission (CR) rate in patients with R/R B-cell acute lymphoblastic leukemia (B-ALL), who received CD19 CAR T-cell therapies, is approximately 90%, while it is only 50% in patients with R/R B-cell NHL (B-NHL) (4–6). Approximately 30% of relapses after CD19 CAR T-cell therapy are characterized by CD19 antigen loss through a variety of mechanisms, including antigen escape or lineage switch (7, 8). Evidence from some studies of solid tumors has shown that compared with single-antigen targeting, dual- or multi-antigen targeting CAR T-cells may result in synergistic effects, which can optimize response rates and prevent antigen escape (9, 10). Like CD19, CD22 is expressed in most B-cell malignancies, which is an effective target for CAR T-cell therapy in B-cell hematological malignancies (11).

As a proto-oncogene, MYC plays a central role in the pathogenesis of DLBCL and is particularly involved in its progression (12). The World Health Organization (WHO) classification considers cases with concurrent MYC and BCL2 and/or BCL6 rearrangement determined by fluorescence *in situ* hybridization (FISH) as “double-hit” lymphomas (DHL) or “triple-hit” lymphomas (THL), respectively. Patients with co-expression of MYC and BCL2 but without underlying rearrangement, as defined by immunohistochemistry (IHC), are commonly referred to as double-expressor (DE) lymphoma (13, 14). Studies suggest that patients with co-expression of double proteins have a higher international prognostic index (IPI) score, advanced-stage disease (III/IV), and poor prognoses (15, 16).

^18^F-FDG PET/CT is already used by clinicians to diagnose and evaluate hematologic malignancies. Recent studies have demonstrated that baseline radiomic features are promising prognostic markers in lymphoma, as they can better predict outcomes compared with conventional imaging metrics (17–19). Radiomics is the high-throughput extraction of a large number of quantitative image features from medical images and can capture information on the intensity, texture, and shape of lesions. Recently, although radiomics has made significant progress in various malignancies (20, 21), radiomic information for R/R B-NHL is still limited.

To the best of our knowledge, no study has introduced textural analysis to predict prognosis in BCL patients treated with CAR T-cell therapies, especially for CD19/CD22-targeting CAR T-cell therapies. Therefore, we aimed to investigate the capacity of radiomic features extracted from baseline^18^F-FDG PET/CT to predict the survival of BCL patients treated with CD19/CD22 dual-targeted CAR T-cell therapies. Moreover, we explored the relationship between baseline radiomic features and the occurrence probability of cytokine release syndrome (CRS).

## Materials and Methods

### Patients

This retrospective study was approved by the institutional review board of the First Affiliated Hospital of Soochow University, and the informed consent was waived. This study was carried out following the Declaration of Helsinki with a trial registration number of ChiCTR2100052247.

The inclusion criteria were set as follows: patients were over 18 years old; patients treated with CD19/CD22 dual-targeted CAR T-cell therapies; and patients who underwent ^18^F-FDG PET/CT before CAR T-cell infusion. Between June 2017 to July 2021, 24 patients with histologically confirmed BCL were enrolled in the present study. CRS was assessed and graded according to the American Society for Transplantation and Cellular Therapy (ASTCT) criteria (22). Neurotoxicity was assessed and graded according to the Common Terminology Criteria for Adverse Events. Cutoff values of 40% for MYC protein and 50% for BCL2 protein were defined as the DE status (13).

### PET/CT Acquisition

The ^18^F-FDG PET/CT examination was performed after 6 h of fasting with blood glucose lower than 11.1 mmol/L. Approximately 40-60 min after the injection of ^18^F-FDG (4.07-5.55 MBq/kg), PET/CT was performed from the base of the skull to the midthigh with 2-3 min per bed position (reconstructed by ordered subset expectation-maximization algorithm) using a Discovery PET/CT (General Electric Medical Systems, Milwaukee WI, USA) with low-dose CT parameters (140 kV, 120 mA, transaxial FOV of 70 cm, slice thickness 3.75 mm).

### Feature Extraction and Selection

**Figure 1:**
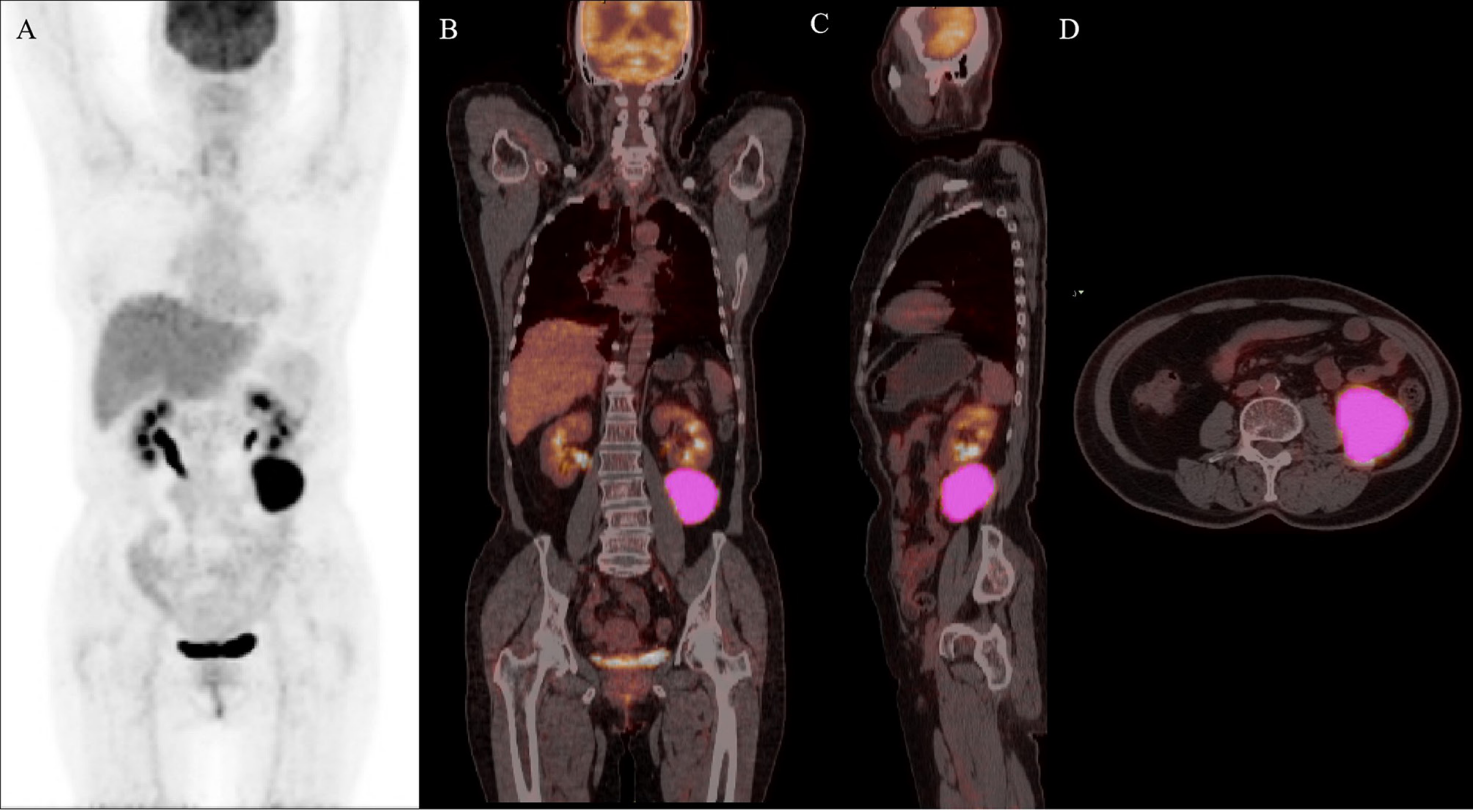
Maximal intensity projection image **(A)**, coronary image **(B)**, sagittal image **(C)**, and transaxial images **(D)** of ^18^F-FDG PET/CT showing an example of VOI for measuring imaging features of BCL. A 68-year-old woman with stage IV DLBCL. MIP image showing metabolically active left abdominal lesion. 3D VOI was manually drawn with the LIFEx segmentation tool using the previously recommended SUV_max_ threshold of 41%.

**Figure 2:**
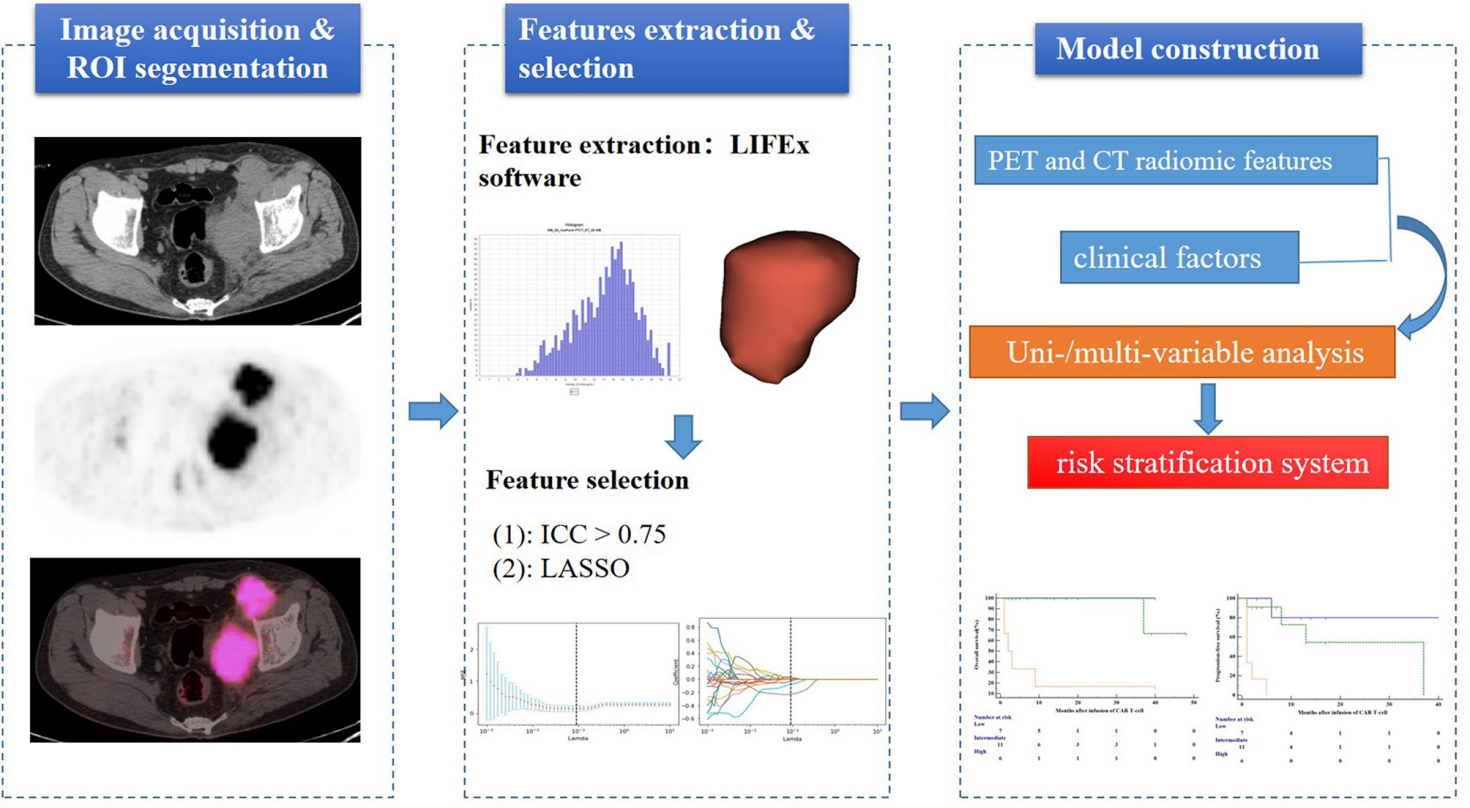
Workflow of the radiomics analysis.

Quantitative PET/CT analysis was performed using the LIFEx freeware (v6.30 https://www.lifexsoft.org/) (23). The volume of interest (VOI) was contoured manually on co-registered images by two experienced nuclear medicine physicians who were blinded to the clinical and pathological information of patients. The whole layers in three-dimensional VOI were delineated on each slice, and 41% of the maximum standardized uptake value (SUV_max_) was used as the threshold to define VOI (24). The details of the tumor segmentation are described in **Figure 1**. Spatial resampling had a voxel size of 2 × 2 × 2 mm. Intensity discretization for CT data was performed with the number of gray levels of 400 bins and absolute scale bounds from -1,000 and 3,000 HU, while it was conducted with 64 bins between 0 and 20 for PET data. The radiomic features were extracted from both PET and CT images within the same VOI due to the good matching of PET and CT images. The radiomics workflow is shown in **Figure 2**. A total of 92 radiomic features were extracted, including 47 PET-derived features and 45 CT-derived features (**Table S1**).

The radiomic features of PET and CT images were selected by the following procedure. Intraclass correlation coefficient (ICC > 0.75) was first performed to remove the redundant features (25, 26). Subsequently, the retained features were further selected by the least absolute shrinkage and selection operator (LASSO) regression algorithm (27). In addition, 10-fold cross-validation was applied to select the parameter of Lambda (λ) to avoid overfitting.

### Statistical Analysis

The categorical variables were analyzed using chi-square statistic or Fisher’s exact test. Mann–Whitney U-test was applied to continuous variables. Progression-free survival (PFS) and overall survival (OS) were defined as the time from first CAR T-cell infusion until disease progression or death from any cause, respectively. The optimal cutoff values for PFS and OS were determined using receiver operating characteristic (ROC) curves according to the Youden index. Survival functions were calculated by the Kaplan-Meier method, and comparisons between subgroups were made using a log-rank test. Univariate and multivariate analyses were carried out using Cox proportional-hazards models. *P* < 0.05 was considered statistically significant. IBM SPSS statistics version 26.0, PYTHON version 3.0 (https://www.python.org), and MedCalc software (MedCalc Software, Ostend, Belgium) were used for statistical analyses.

## Results

### Patient Characteristics

A total of 24 patients (median age of 51 years, range of 26-70 years) who received CAR T-cell therapy were enrolled in the present study. **Table 1** summarizes the baseline characteristics of the patients. For the histological type, 20 (83.33%) patients had DLBCL, two (8.33%) had transformed follicular lymphoma, one (4.17%) had B-cell lymphoblastic lymphoma, and one (4.17%) had Burkitt lymphoma. The median number of prior therapies was 2 (range of 1-5). Eight (33.33%) patients underwent prior autologous hematopoietic stem cell transplantation (HSCT).

**Table 1:** Patient baseline characteristics.

Characteristics	No. of patients (n = 24)
Male gender	16 (66.67%)
Median age (range), y	51 (26-70)
Ann Arbor stage (at diagnosis)	
II	3 (12.50%)
III	5 (20.83%)
III	5 (20.83%)
IV	16 (66.67%)
B symptom (yes)	10 (41.67%)
Lymphoma subtype	
DLBCL	20 (83.33%)
BL	1 (4.17%)
trFL	2 (8.33%)
B-LBL	1 (4.17%)
LDH > UNL	8 (33.33%)
ECOG ≥ 2	3 (12.50%)
Marrow involvement (+)	9 (37.50%)
Number of prior therapies median (range)	2 (1-5)
Prior HSCT (yes)	8 (33.33%)
IPI (at diagnosis) ≥ 3	15 (62.50%)
BCL2/MYC double expression	11 (45.83%)
BCL2 expression	18 (75.00%)
MYC expression	12 (50.00%)

diffuse large B-cell lymphoma (DLBCL); Burkitt lymphoma (BL); transformed follicular lymphoma (trFL); B-cell lymphoblastic lymphoma (B-LBL); lactate dehydrogenase (LDH); upper limit of normal (ULN); Eastern Cooperative Oncology Group (ECOG); Autologous stem cell transplant (ASCT); International Prognostic Index (IPI).

**Figure 3:**
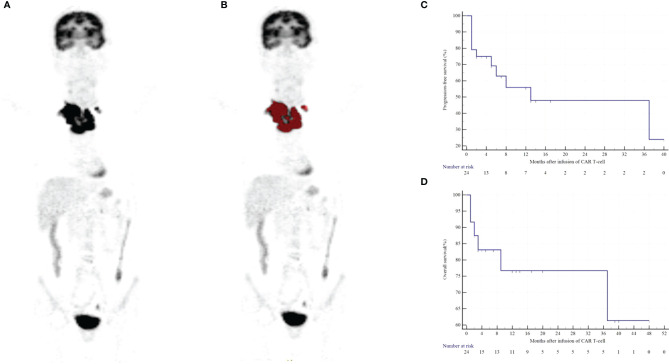
Clinical outcomes of the BCL patients following the infusion of CD19/CD22 dual-targeted CAR T-cells. A patient with a high-risk NGLDM_Contrast_PET_ (> 0.468) and positive-DE showed progression after 5 months and died 9 months after CAR T-cell therapy **(A, B)**. Kaplan-Meier curves of PFS **(C)** and OS **(D)**.

The median OS for the entire group was not reached. OS rates at 1 and 2 years were 79.17% and 79.17%, respectively (**Figure 3D**). The median PFS was 13 months. The 1- and 2-year PFS rates were 58.33% and 58.33%, respectively (**Figure 3C**). Six patients died after infusion, with a median time of 2.5 months (range of 1-37 months), and all deaths were attributed to the progression of lymphoma (**Figures 3A, B**). Moreover, 11 patients relapsed/progressed at a median time of 2 months (range of 6 to 37) after infusion.

### Feature Selection

A total of 92 radiomic features were extracted from PET and CT images. Based on the LASSO regression model, we obtained five and four radiomic features for PFS and OS, respectively. For PFS, the CT zone percentage from gray-level zone-length matrix (GLZLM_ZP_CT_), PET run-length non-uniformity from gray-level run-length matrix (GLRLM_GLNU_PET_), Contrast from neighborhood gray-level difference matrix (NGLDM_Contrast_PET_), SHAPE_Volume_PET_, and SHAPE_Sphericity (SHAPE_Sphericity_PET_) were selected. For OS, CT long-zone high gray-level emphasis from gray-level zone-length matrix (GLZLM_LZHGE_CT_), PET Energy from grey-level co-occurrence matrix (GLCM_Energy_PET_), NGLDM_Contrast_PET_, and zone-length non-uniformity from gray-level zone-length matrix (GLZLM_ZLNU_PET_) were selected. The ICC of the radiomic features was all above 0.75. **Table 2** summarizes the ROC analysis results of PFS and OS.

**Table 2:** ROC analyses for PFS and OS.

	PFS						OS				
	AUC	cutoff	p	Se (%)	Sp (%)		AUC (95%CI)	cutoff	p	Se (%)	Sp (%)
GLZLM_ZP_CT_	0.769 (0.577-0.962)	≤ 0.337	0.006	100	53.8	GLZLM_LZHGE_CT_	0.620 (0.376-0.865)	>721377.442	0.334	100	38.89
SHAPE_Volume_PET_	0.538 (0.294-0.783)	> 70.000	0.758	100	30.77	GLCM_Energy_PET_	0.556 (0.271-0.840)	> 0.001	0.702	100	22.2
GLRLM_GLNU_PET_	0.573 (0.328-0.819)	≤ 40.495	0.558	100	38.5	NGLDM_Contrast_PET_	0.898 (0.769-1.000)	> 0.473	< 0.00 01	100	72.2
NGLDM_Contrast_PET_	0.930 (0.834-1.000)	> 0.468	< 0.0001	90.91	84.62	GLZLM_ZLNU_PET_	0.574 (0.343-0.805)	> 16.294	0.530	100	33.3
SHAPE_Sphericity_PET_	0.783 (0.593-0.973)	> 0.467	0.0035	100	53.85	MTV	0.644 (0.410-0.877)	> 42.000	0.2276	83.33	61.11
MTV	0.738 (0.524-0.952)	> 35.500	0.0294	81.82	69.23	TLG	0.667 (0.449-0.885)	> 55.000	0.1340	100	44.44
TLG	0.783 (0.574-0.992)	>55.000	0.0079	100	61.54						

AUC: area under the receiver operating characteristic curve; Se: Sensitivity; Sp: Specificity.

### Characteristics and Outcomes

**Figure 4:**
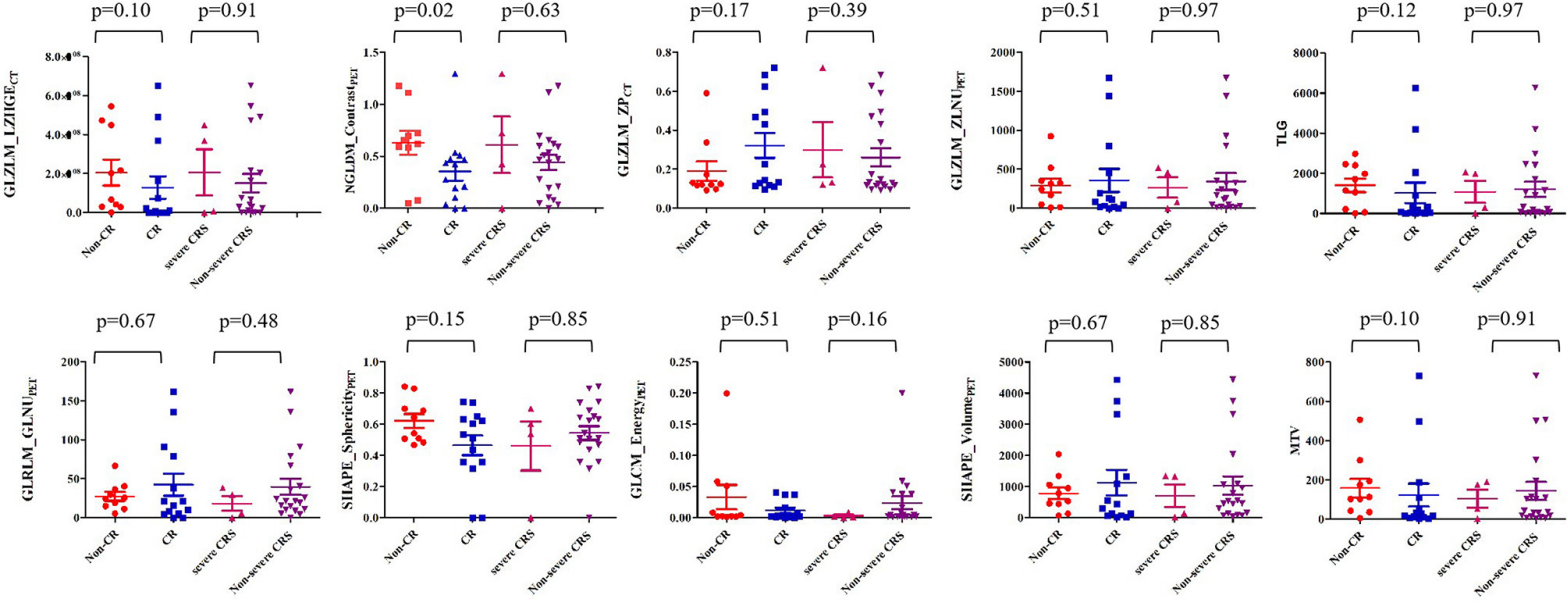
Comparison of textural features between non-CR and CR patients and between severe CRS and non-severe CRS patients.

After a median follow-up of 10.5 months (range of 1-48 months), the overall response (OR) rate, evaluated by ^18^F-FDG PET/CT or CT at 1 month after infusion, was 87.5% (21/24), 50% (14/24) patients achieved CR, and 29.17% (7/24) patients achieved partial response (PR). The median NGLDM_Contrast_PET_ was 0.350 (range of 0.000-1.296) in patients who achieved CR, which was significantly lower compared with patients who did not achieve CR (median 0.635 [range of 0.046-1.177]; p= 0.02; **Figure 4**). Patients who received less than two lines of chemotherapy had a higher probability of CR (p= 0.03). No significant association was found between other characteristics and outcomes in this cohort (all p>0.089, **Tables S2** and **S3**).

### Univariable and Multivariate Analyses

**Figure 5:**
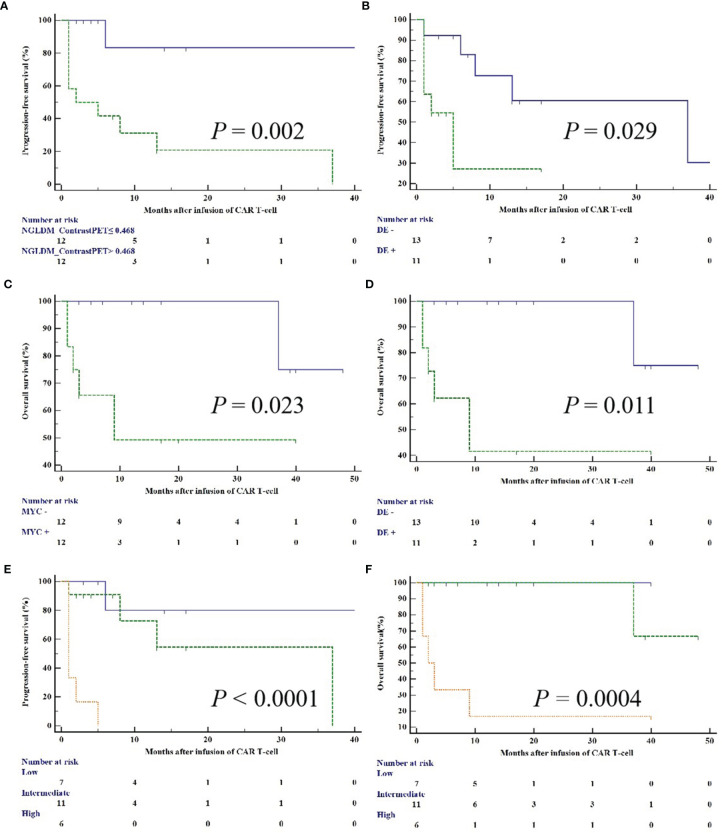
Kaplan–Meier curves for PFS and OS according to NGLDM_Contrast_PET_ **(A)**, MYC and BCL2 DE **(B, D)**, MYC **(C)**, and PET/CT scoring system **(E, F)**.

We performed univariable analysis on survival predictors. LDH, International Prognosis Index (IPI) at diagnosis, prior lines of chemotherapy, response, DE, MYC expression, metabolic tumor volume (MTV), total lesion glycolysis (TLG), GLZLM_ZP_CT_, NGLDM_Contrast_PET_, and SHAPE_Sphericity_PET_ were associated with PFS (**Table 3**), whereas DE, MYC expression, MTV, and NGLDM_Contrast_PET_ were associated with OS (**Table 4**). Patients with high NGLDM_Contrast_PET_ (> 0.468) had a PFS of 16.7%, while patients with low NGLDM_Contrast_PET_ (≤ 0.468) had a PFS of 91.7% (p = 0.002, HR = 6.92, 95%CI = 1.08-15.24, **Figure 5A**). The PFS rates of the negative-DE and positive-DE groups were 61.5% and 45.5%, respectively (p = 0.029, HR = 5.36, 95%CI = 1.19-24.19, **Figure 5B**).

**Table 3:** Univariable and multivariate analyses of predictive factors for PFS.

Variable	Univariable		Multivariate	
	HR (95%CI)	P	HR (95%CI)	P
Female	0.41 (0.11-1.59)	0.198		
Ann Arbor stage III-IV	3.71 (0.64-21.56)	0.144		
B symptom	3.64 (0.92-14.43)	0.066		
LDH > ULN	11.39 (2.29-56.75)	0.003*		0.299
ECOG ≥ 2	1.43 (0.24-8.52)	0.697		
Extranodal sites ≥1	0.64 (1.13-19.83)	0.500		
IPI 3-5	4.06 (1.08-15.24)	0.038*		
Marrow involvement	0.97 (0.26-3.67)	0.964		
Prior lines of chemotherapy>2	4.36 (1.07-17.75)	0.040*		0.197
Prior ASCT	0.82 (0.17-3.93)	0.800		
Grade of CRS 3-4	3.53 (0.57-21.62)	0.173		
Response Non-CR	6.67 (1.63-27.37)	0.009*		0.227
DE	5.36 (1.19-24.19)	0.029*	7.02 (1.16-42.45)	0.047*
MYC +	6.50 (1.52-27.72)	0.012*		0.054
BCL2 +	1.79 (0.45-7.08)	0.404		
MTV> 35.500 cm^3^	12.30 (2.99-50.57)	0.001*		0.106
TLG>55.000	6.13 (1.72-22.20)	0.005*		0.110
GLZLM_ZP_CT_ ≤ 0.337	0.18 (0.05-0.67)	0.010*		0.056
SHAPE_Volume_PET_> 70.000	3.87 (0.74-20.31)	0.110		
GLRLM_GLNU_PET_≤ 40.495	0.25 (0.05-1.40)	0.115		
NGLDM_Contrast_PET_> 0.468	6.92 (1.08-15.24)	0.002*	15.16 (1.77-129.48)	0.023*
SHAPE_Sphericity_PET_> 0.467	4.74 (1.13-19.83)	0.033*		0.068

*p < 0.05.

**Table 4:** Univariable and multivariate analyses of predictive factors for OS.

	Univariable	Multivariate		Univariable
Variable	95%CI	P	Variable	95%CI
Female	0.70 (0.13-3.71)	0.676		
Ann Arbor stage III-IV	1.12 (0.13-9.66)	0.921		
B symptom	1.84 (0.34-9.87)	0.477		
LDH>ULN	2.94 (0.49-17.76)	0.239		
ECOG ≥ 2	0.26 (0.032-2.14)	0.211		
Extranodal sites ≥1	0.68 (0.13-3.61)	0.648		
IPI 3-5	3.00 (0.57-15.64)	0.193		
Marrow involvement	0.96 (0.17-5.36)	0.966		
Prior lines of chemotherapy>2	1.81 (0.34-9.71)	0.490		
Prior ASCT	0.26 (0.04-1.66)	0.153		
Grade of CRS 3-4	8.29 (0.66-103.30)	0.100		
Response Non-CR	3.02 (0.58-15.61)	0.187		
DE	9.56 (1.69-53.99)	0.011*	10.37 (1.17- 92.25)	0.041*
MYC +	7.17 (1.32-38.99)	0.023*	8.64 (0.96- 77.44)	0.042*
BCL2 +	4.30 (0.73-25.37)	0.107		
MTV> 42.000	6.25 (1.171-33.33)	0.032*		0.207
TLG> 55.000	4.49 (0.78-25.79)	0.092		
GLZLM_LZHGE_CT_>721377.442	4.27 (0.72-25.29)	0.110		
GLCM_Energy_PET_> 0.001	3.43 (0.33-35.45)	0.301		
NGLDM_Contrast_PET_> 0.473	9.24 (1.81-47.15)	0.008*		0.196
GLZLM_ZLNU_PET_> 16.294	3.83 (0.58-25.22)	0.162		

*p < 0.05.

For multivariate analysis, DE (HR = 7.02; 95%CI = 1.16-42.45; p = 0.047) and NGLDM_Contrast_PET_ (HR =15.16; 95%CI =1.77-129.48; p = 0.023) were two independent prognostic factors associated with PFS. DE (HR = 10.37; 95%CI = 1.17- 92.25; p = 0.041) and MYC expression (HR = 8.64; 95%CI = 0.96- 77.44; p = 0.042) were prognostic factors for OS (**Figures 5C, D**).

### Prognostic Model Construction

A clinical model was constructed based on multivariate Cox analysis of significant risk factors. The risk factors included higher NGLDM_Contrast_PET_ (>0.468) and positive DE. The clinical and PET models were combined, and all patients were stratified into three risk categories with distinct PFS (p < 0.0001) and OS (p = 0.0004): group I (no risk factors; n = 7); group II (one risk factor only; n = 11); and group III (two risk factors; n = 6). These three groups had significantly different PFS, which was 85.7% (group I), 63.6% (group II), and 0% (group III), respectively, (group I *vs.* group II: HR = 1.206 and p =0.272; group I *vs.* group III: HR = 13.791 and p = 0.0002; group II *vs.* group III: HR = 13.788 and p = 0.0002; **Figure 5E**). They also had a significantly different OS, which was 100% (group I), 90.9% (group II), and 16.7% (group III), respectively, (group I *vs.* group II: HR=0.333 and p=0.564; group I *vs.* group III: HR=8.884 and p=0.003; group II *vs.* group III: HR=9.239 and p=0.002; **Figure 5F**).

### Baseline Variables and Adverse Effects

In our present study, 14 patients (58%) experienced CRS, including seven (29%) patients with Grade 1 CRS, three (13%) patients with Grade 2 CRS, and four (16%) patients with Grade 3 CRS. All patients responded to established conventional therapies, and no patients received tocilizumab treatment. The PET/CT variables in patients with non-severe CRS (0-2) were not significantly different from those who had severe CRS (3-4) (all p > 0.05, **Figure 4** and **Table S4**). No patient developed neurotoxicity.

## Discussion

In the current study, we investigated the potential prognostic value of radiomic features derived from ^18^F-FDG PET/CT images in BCL patients treated with CD19/CD20-targeting CAR T-cell therapies. The results showed that DE and NGLDM_Contrast_PET_ were two independent prognostic factors for PFS, whereas DE and MYC expression were of prognostic significance for OS. Moreover, a prognostic stratification model was established to identify risk stratification of patients by integrating clinicopathological and PET/CT imaging prognostic factors. Our findings indicated that the baseline PET/CT-based radiomic features might contribute to the risk stratification of BCL patients.

Radiomics is an emerging method to acquire a large amount of high-dimensional data through the analysis of entire disease lesions and can quantify the non-uniformity between adjacent voxels. Measuring the textural indices of PET/CT images to quantify intratumor heterogeneity has been proposed as an adjunct to predict outcomes (17, 18, 20). Moreover, due to the differences in radiomic features and numbers extracted, it is difficult to make direct comparisons between studies. Lue et al. (17) have found that baseline PET-radiomic feature (GLRLM_RLN) can be used to predict the survival outcomes of DLBCL. Cheng et al. (28) have suggested that uniformity from NGLCM is a significant prognostic factor for patients with oropharyngeal squamous cell carcinoma. To the best of our knowledge, this was the first report to investigate the potential of FDG PET/CT radiomic features for outcome prediction in BCL patients treated with CAR T-cell therapies. To reduce the impact of discretization values on robustness, an absolute resampling was used. Several radiomic features extracted from PET and CT images were prognostic factors: including GLZLM_ZP_CT_, GLRLM_GLNU_PET_, NGLDM_Contrast_PET_, SHAPE_Volume_PET_, SHAPE_Sphericity_PET_, GLZLM_LZHGE_CT_, GLCM_Energy_PET_, and GLZLM_ZLNU_PET_. Finally, the multivariable analysis revealed that Contrast extracted from NGLDM appeared to be the independent predictor of PFS. NGLDM is expressed as grey-level variability between one voxel and its neighbors in three dimensions. Higher NGLDM_Contrast_PET_ was associated with poor prognosis, indicating the importance of tumor heterogeneity on PET as a predictor of disease progression.

Genetic rearrangements of MYC and BCL2 and lymphoma derived from MYC and BCL2 co-expression have received increasing attention in recent years (29). DE lymphoma accounts for 18% to 42% of DLBCL cases, which may be related to gene amplification or translocation (29–31). In our present study, 45.8% of the analyzed patients demonstrated co-expression of MYC and BCL2 proteins. Similarly, in a study by Xu et al. (31), 42% of the patients show these aberrations. Besides, co-expression of MYC and BCL2 is a prognostic biomarker in the management of DLBCL (32). Recent data have demonstrated that patients with DE are more likely to have high-risk gene signatures and more inferior prognoses (33, 34). These findings emphasize the value of DE as a promising tool for prognostic predictors. Compared with fluorescence *in situ* hybridization (FISH), co-expression of MYC and BCL2 defined by IHC assay represents an inexpensive, rapid, and reproducible technique that has broader application prospects in clinical practice.

In the present study, NGLDM_Contrast_PET_ and DE had independent predictive values. We suggested establishing a prognostic scoring system based on these two characteristics that were complementary in that sense, and they were characterized by two different aspects of the disease: radiomic features and genomic features. This score appeared to have a higher capability for patient risk stratification. Combination of NGLDM_Contrast_PET_ and DE stratified the population into three different prognostic groups; group I (low NGLDM_Contrast_PET_ and negative-DE; PFS 85.7%, OS 100%), group II (high NGLDM_Contrast_PET_ or positive-DE; PFS 63.6%, OS 90.9%), and group III (high NGLDM_Contrast_PET_ and positive-DE; PFS 0%, OS 16.7%). Our results suggested that the combination of these two parameters could provide promising prognostic information for B-NHL patients.

Few studies have investigated the role of MTV in predicting treatment outcomes in BCL (35–37). In lymphoma, several reports have also indicated that PET/CT radiomic features are significantly associated with survival, whereas conventional PET metabolic parameters (SUV, MTV, and TLG) are not (17, 38). In another study with NHL patients treated with CD19-targeting CAR T-cell therapy, TMTV does not have a significant association with OS (39). In contrast, Wang et al. (40) have reported that traditional imaging parameters are more efficient than textural features for predicting therapeutic response and survival. Dean et al. (41) have reported that baseline TMTV, in large B-cell lymphoma (LBCL) patients who are treated with axicabtagene ciloleucel (axi-cel), has a significant prognostic impact on PFS and OS. In our present study, MTV was a prognosticator of both PFS and OS in the univariate analysis. However, we noticed that the statistical significance was not achieved in the multivariate analysis, which was presumably attributed to the small sample size. Many factors may contribute to these differences, such as small sample size, reconstruction parameters, segmentation, and software (25). Our results indicated that the features of intratumor heterogeneity might serve as a complementary indicator and outperform MTV. Further studies are required in a larger cohort population to validate our findings.

In agreement with the previous studies (42), we did not observe any association between PET/CT parameters and CRS, possibly because of the relatively limited number of patients included and the small number of high-grade CRS (≥2) in patients receiving CD19/CD22 CAR T-cell therapies.

There are several limitations to our study. First, it lacked a patient cohort for external validation, and these results should be validated in a large cohort of patients. Second, the number of patients included in this study was relatively small due to the novelty of the therapy. Other limitations of the study derived from the single-center and retrospective data.

## Conclusions

In conclusion, radiomic features extracted from baseline^18^F-FDG PET/CT images in combination with genomic factors could predict the survival outcomes of BCL patients receiving CAR T-cell therapy. No significant association was found between PET/CT parameters and CRS. Further prospective studies and validation in a large cohort of patients are required to confirm these results.

## Data Availability Statement

The datasets presented in this study can be found in online repositories. The names of the repository/repositories and accession number(s) can be found in the article/**Supplementary Material**.

## Ethics Statement

The studies involving human participants were reviewed and approved by the First Affiliated Hospital of Soochow University. Written informed consent for participation was not required for this study in accordance with the national legislation and the institutional requirements.

## Author Contributions

YZ, JL, XZ, and SD conceptualized and designed the study. TJ, BZ, ND, and SS performed analysis. SS and SD interpreted the data. YZ and JL drafted the manuscript. SS and SD revised the manuscript. All authors contributed to the article and approved the submitted version.

## Funding

This research was funded by the National Natural Science Foundation of China (81601522), Medical Youth Talent Project of Jiangsu Province (QNRC2016749), Gusu Health Talent Program (GSWS2020013), Suzhou People’s Livelihood Science and Technology Project (SYS2019038), Project of State Key Laboratory of Radiation Medicine and Protection, Soochow University (GZK1202127), and the open Foundation of Nuclear Medicine Laboratory of Mianyang Central Hospital, (2021HYX023 and 2021HYX029).

## Conflict of Interest

The authors declare that the research was conducted in the absence of any commercial or financial relationships that could be construed as a potential conflict of interest.

## Publisher’s Note

All claims expressed in this article are solely those of the authors and do not necessarily represent those of their affiliated organizations, or those of the publisher, the editors and the reviewers. Any product that may be evaluated in this article, or claim that may be made by its manufacturer, is not guaranteed or endorsed by the publisher.
