# Supplementary material for: Prognostic Value of Radiomic Features of 18F-FDG PET/CT in Patients With B-Cell Lymphoma Treated With CD19/CD22 Dual-Targeted Chimeric Antigen Receptor T Cells
Source: Front Oncol. 2022 Feb 7;12:834288. doi: 10.3389/fonc.2022.834288 (PMC8858981; doi:10.3389/fonc.2022.834288)
Supplement: Supplementary file 1 [file Table_1.docx]

**Table S1 Textural features calculated from the CT and PET images.**

| **Conventional textural features** | **First-order textural features** |
| --- | --- |
| SUVmin  SUVmean  SUVstd  SUVmax  SUVpeak*  TLG* | HISTO_Skewness  HISTO_Kurtosis  HISTO_Entropy_log10  HISTO_Entropy_log2  HISTO_Energy  SHAPE_Sphericity  SHAPE_Compacity  SHAPE_Volume (mL)  SHAPE_Volume(vx) |
| **Higher-order textural features** | |
| **GLZLM** | **GLRLM** |
| GLZLM_SZE (Short-Zone Emphasis)  GLZLM_LZE (Long-Zone Emphasis)  GLZLM_LGZE (Low Gray-level Zone Emphasis)  GLZLM_HGZE (High Gray-level Zone Emphasis)  GLZLM_SZLGE (Short-Zone Low Gray-level Emphasis)  GLZLM_SZHGE (Short-Zone High Gray-level Emphasis)  GLZLM_LZLGE (Long-Zone Low Gray-level Emphasis)  GLZLM_LZHGE (Long-Zone High Gray-level Emphasis)  GLZLM_GLNU (Gray-Level Non-Uniformity for zone)  GLZLM_ZLNU (Zone Length Non-Uniformity)  GLZLM_ZP (Zone Percentage) | GLRLM_SRE (Short-Run Emphasis)  GLRLM_LRE (Long-Run Emphasis)  GLRLM_LGRE (Low Gray-level Run Emphasis)  GLRLM_HGRE (High Gray-level Run Emphasis)  GLRLM_SRLGE (Short-Run Low Gray-level Emphasis)  GLRLM_SRHGE (Short-Run High Gray-level Emphasis)  GLRLM_LRLGE (Long-Run Low Gray-level Emphasis)  GLRLM_LRHGE (Long-Run High Gray-level Emphasis)  GLRLM_GLNU (Gray-Level Non-Uniformity for run)  GLRLM_RLNU (Run Length Non-Uniformity)  GLRLM_RP (Run Percentage) |
| **GLCM** | **NGLDM** |
| GLCM_Homogeneity  GLCM_Energy  GLCM_Contrast  GLCM_Correlation  GLCM_Entropy_log10  GLCM_Entropy_log2  GLCM_Dissimilarity | NGLDM_Coarseness  NGLDM_Contrast  NGLDM_Busyness |

* Calculated only for PET

**Table S2 Relationships between outcomes and clinical characteristics of patients**

| **Characteristic** |  | **Total population (n=24)** | **CR (n=14)** | **NCR (n=10)** | **P** |
| --- | --- | --- | --- | --- | --- |
| Sex | Male | 16 (66.67%) | 8 (57.14%) | 8 (80.00%) | 0.39 |
|  | Female | 8 (33.33%) | 6 (42.86%) | 2 (20.00%) |  |
| Ann Arbor stage (at diagnosis) | I-II | 3 (12.50%) | 3 (21.43%) | 0 (0.00%) | 0.24 |
|  | III-IV | 21(87.50%) | 11 (78.57%) | 10 (100.00%) |  |
| B symptom | Yes | 9 (37.50%) | 4 (28.57%) | 5 (50.00%) | 0.40 |
|  | No | 15 (62.50%) | 10 (71.43%) | 5 (50.00%) |  |
| LDH | > UNL | 8 (33.33%) | 4 (28.57) | 4 (40.00%) | 0.67 |
|  | Normal | 16 (66.67%) | 10 (71.43%) | 6 (60.00%) |  |
| ECOG | 0-1 | 21 (87.50%) | 12 (85.71%) | 9 (90.00%) | 0.63 |
|  | ≥ 2 | 3 (12.50%) | 2 (14.29%) | 1(10.00%) |  |
| Extranodal sites | Yes | 12 (50.00%) | 7(50.00%) | 5 (50.00%) | 0.66 |
|  | No | 12 (50.00%) | 7 (50.00%) | 5 (50.00%) |  |
| IPI at diagnosis | 0-1 | 9 (37.50%) | 7 (50.00%) | 2 (20.00%) | 0.21 |
|  | ≥ 3 | 15 (62.50%) | 7 (50.00%) | 8 (80.00%) |  |
| Marrow involvement | + | 9 (37.50%) | 6 (42.86%) | 3 (30.00%) | 0.68 |
|  | - | 15 (62.50%) | 8 (57.14%) | 7 (70.00%) |  |
| Number of prior therapies | > 2 | 8 (33.33%) | 2 (14.29%) | 6 (60.00%) | 0.03* |
|  | ≤ 2 | 16 (66.67%) | 12 (85.71%) | 4 (40.00%) |  |
| Prior ASCT | Yes | 6 (25.00%) | 3 (21.43%) | 3 (30.00%) | 0.67 |
|  | No | 18 (75.00%) | 11 (78.57%) | 7 (70.00%) |  |
| Grade of CRS | 0-2 | 20 (83.33%) | 11 (78.57%) | 9 (90.00%) | 0.62 |
|  | 3-4 | 4 (16.67%) | 3 (21.43%) | 1 (10.00%) |  |
| DE | + | 11 (45.83%) | 5 (35.71%) | 6 (60.00%) | 0.41 |
|  | - | 13 (54.17%) | 9 (64.29%) | 4 (40.00%) |  |
| MYC expression | + | 12 (50.00%) | 6 (42.86%) | 6 (60.00%) | 0.41 |
|  | - | 12 (50.00%) | 8 (57.14%) | 4 (40.00%) |  |
| BCL-2 expression | + | 18 (75.00%) | 11 (78.57%) | 7 (70.00%) | 0.67 |
|  | - | 6 (25.00%) | 3 (21.43%) | 3 (30.00%) |  |

*P value < 0.05.

LDH, lactate dehydrogenase; ECOG, Eastern Cooperative Oncology Group; HSCT, autologous hematopoietic stem cell transplantation; IPI, International Prognostic Index.

**Table S3 Relationships between outcomes and PET/CT paraments of patients**

| **Characteristic** | **Total population (**Mean±SD**)** | **CR (**Mean±SD**)** | **NCR (**Mean±SD**)** | **P** |
| --- | --- | --- | --- | --- |
| MTV | 47.50±190.56 | 24.50±219.30 | 108.00±150.11 | 0.10 |
| TLG | 299.00±1594.61 | 134.00±1907.73 | 1427.50±1073.35 | 0.12 |
| GLZLM_LZHGE_CT_ | 35650000.00±212371177.15 | 8557928.63±215823481.29 | 134250000.00±210000686.94 | 0.10 |
| GLZLM_ZLNU_PET_ | 149.11 | 95.24 | 277.71 | 0.51 |
| GLCM_Energy_PET_ | 0.003±0.0421 | 0.003±0.015 | 0.003±0.062 | 0.51 |
| GLZLM_ZP_CT_ | 0.13±0.22 | 0.19±0.24 | 0.12±0.16 | 0.17 |
| SHAPE_Volume_PET_ | 496.00±1226.03 | 373.00±1536.53 | 660.50±589.71 | 0.67 |
| GLRLM_GLNU_PET_ | 21.41±42.16 | 18.57±53.19 | 24.99±17.60 | 0.67 |
| NGLDM_Contrast_PET_ | 0.47±0.37 | 0.35±0.33 | 0.64±0.36 | 0.02* |
| SHAPE_Sphericity_PET_ | 0.54±0.21 | 0.52±0.24 | 0.59±0.14 | 0.15 |

*P value < 0.05.

**Table S4 Relationships between CRS and PET/CT paraments of patients**

| **Characteristic** | **Total population (**Mean±SD**)** | severe CRS  **(**Mean±SD**)** | non-severe CRS **(**Mean±SD**)** | **P** |
| --- | --- | --- | --- | --- |
| MTV | 47.500±190.56 | 114.00±91.78 | 38.758±205.82 | 0.91 |
| TLG | 299.00±1594.61 | 1130.500±1083.14 | 262.00±1700.03 | 0.97 |
| GLZLM_LZHGE_CT_ | 35650000.00±212371177.15 | 187807928.63±236490186.04 | 35650000.00±212667509.63 | 0.91 |
| GLZLM_ZLNU_PET_ | 149.11±453.47 | 267.81±262.02 | 149.11±486.82 | 0.97 |
| GLCM_Energy_PET_ | 0.003±0.042 | 0.002±0.004 | 0.004±0.045 | 0.16 |
| GLZLM_ZP_CT_ | 0.13±0.22 | 0.18±0.29 | 0.13±0.21 | 0.39 |
| SHAPE_Volume_PET_ | 496.00±1226.03 | 732.00±722.48 | 496.00±1310.87 | 0.85 |
| GLRLM_GLNU_PET_ | 21.41±42.16 | 17.47±18.48 | 21.41±44.92 | 0.48 |
| NGLDM_Contrast_PET_ | 0.47±0.37 | 0.57±0.54 | 0.47±0.33 | 0.63 |
| SHAPE_Sphericity_PET_ | 0.54±0.21 | 0.57±0.31 | 0.53±0.20 | 0.85 |
